# Biochemical Characterization of Phenylacetaldehyde Dehydrogenases from Styrene-degrading Soil Bacteria

**DOI:** 10.1007/s12010-020-03421-8

**Published:** 2020-10-27

**Authors:** Juliane Zimmerling, Michel Oelschlägel, Carolin Großmann, Matthias Voitel, Michael Schlömann, Dirk Tischler

**Affiliations:** 1 Interdisciplinary Ecological Center, Environmental Microbiology Group, TU Bergakademie Freiberg, Leipziger Str. 29, 09599 Freiberg, Germany; 2 Microbial Biotechnology, Faculty of Biology and Biotechnology, Ruhr-Universität Bochum, Universitätsstr. 150, 44780 Bochum, Germany

**Keywords:** Oxidoreductase, NAD^+^, Maximum reaction rate, Michaelis constant, Turnover number, Evolutionary ancestry, Esterase activity

## Abstract

Four phenylacetaldehyde dehydrogenases (designated as FeaB or StyD) originating from styrene-degrading soil bacteria were biochemically investigated. In this study, we focused on the Michaelis-Menten kinetics towards the presumed native substrate phenylacetaldehyde and the obviously preferred co-substrate NAD^+^. Furthermore, the substrate specificity on four substituted phenylacetaldehydes and the co-substrate preference were studied. Moreover, these enzymes were characterized with respect to their temperature as well as long-term stability. Since aldehyde dehydrogenases are known to show often dehydrogenase as well as esterase activity, we tested this capacity, too. Almost all results showed clearly different characteristics between the FeaB and StyD enzymes. Furthermore, FeaB from *Sphingopyxis fribergensis* Kp5.2 turned out to be the most active enzyme with an apparent specific activity of 17.8 ± 2.1 U mg^-1^. Compared with that, both StyDs showed only activities less than 0.2 U mg^-1^ except the overwhelming esterase activity of StyD-CWB2 (1.4 ± 0.1 U mg^-1^). The clustering of both FeaB and StyD enzymes with respect to their characteristics could also be mirrored in the phylogenetic analysis of twelve dehydrogenases originating from different soil bacteria.

**Electronic supplementary material:**

The online version of this article (10.1007/s12010-020-03421-8) contains supplementary material, which is available to authorized users.

## Introduction

Aldehyde dehydrogenases (ALDHs, EC 1.2.1) comprise a huge group of enzymes occurring in every organism ranging from bacteria and fungi to invertebrates and mammals [1]. Enzymes belonging to this class transform a wide range of substrates during different metabolic routes, and they are strictly co-substrate-dependent. The substrate spectra of these enzymes comprise aldehydes which are oxidized to acids while reducing NAD(P)^+^.

By means of the 145 aligned ALDHs, ten conserved motifs could be detected [1]. Four of them were allocated to the co-substrate binding region and three to the catalytic site. On the basis of the three catalytic amino acids cysteine, glutamate and asparagine, the catalytic mechanism of ALDHs can be explained as follows. The three amino acids mentioned above form in general a catalytic triad supporting the redox catalysis. The thiol group of cysteine is activated via deprotonation by the glutamate residue and then binds temporarily to the carbonyl C of the substrate. Thereby, it supports the elimination of a hydride from the carbonyl C. The hydride mentioned is directly transferred to NAD(P)^+^ and NAD(P)H + H^+^ is released while the proton originates from glutamate. Subsequently, glutamate deprotonates a water molecule in the next step and a hydroxide ion is formed. This ion can attack the carbonyl C as a nucleophile leading to an organic acid as product. During this transformation, asparagine stabilizes the carbonyl O and the meanwhile occurring oxyanion [2, 3]. The catalytic triad mentioned above is also responsible for the often observed esterase activity of these ALDHs.

In this above-mentioned study of 1999, 145 ALDH sequences were aligned. On the basis of this alignment, a phylogenetic tree was constructed which is subdivided into two main trunks [1]. One main trunk consists of class 3 ALDHs (eight families), the other one of class 1 (one family), and 2 ALDHs (five families). Class 1 and 2 ALDHs have various substrate spectra except for two enzyme families. In contrast, nearly all class 3 ALDH representatives are very substrate-specific and occur as homotetramer [2, 4]. Perozich and coworkers [4] proposed that the substrate-unspecific enzymes diverged later in evolution than the substrate-specific ALDHs. The substrate specificity likely depends on the aim which is pursued in the metabolic context. Basic cellular metabolic pathways require substrate-specific biocatalysts, whereas enzymes with unspecific substrate spectra are most suitable to metabolize xenobiotics. Maybe, this is a strategy to occupy ecological niches. Furthermore, nearly each family has a clear co-substrate preference, and the majority of these enzymes belonging to the same families occurred from bacteria to humans. Nevertheless, three families were only found in certain kingdoms like the aromatic, fungal and class 1 ALDHs. The last-mentioned ones were only detected in animals, and the aromatic ALDHs were found exclusively in bacteria [1].

In our study, we focused on bacterial ALDHs, especially phenylacetaldehyde dehydrogenases (PADs) originating from the upper pathway of the microbial styrene metabolism. The aerobic styrene degradation can be divided into two routes, the side-chain oxygenation and the direct ring cleavage [5]. The side-chain oxygenation consists of the upper and the lower pathway. Three enzymes operate sequentially in the upper styrene catabolic pathway. First, the styrene monooxygenase (SMO) forms the reactive styrene oxide which is then isomerized to phenylacetaldehyde by the styrene oxide isomerase (SOI). Now, the aldehyde is oxidized by a PAD to phenylacetic acid [6]. Phenylacetic acid product of the upper styrene catabolic pathway is transformed into acetyl-CoA and succinyl-CoA through the following enzymatic transformations in the lower styrene catabolic pathway [7].

So far, especially SMOs and SOIs of different microorganisms were studied in detail [8]. The PADs studied, so far, originate from the bacteria *Pseudomonas fluorescens* ST [9], *Pseudomonas putida* S12 [2], *Escherichia coli* K-12 [10–13], *Escherichia coli* ATCC 11105 W [10], *Arthrobacter globiformis* [14], *Xanthobacter* sp. 124X [15], *Rhodococcus rhodochrous* [6], and the fungus *Exophiala jeanselmei* [16]. In contrast to SMOs and SOIs, the PADs have been less extensively characterized in terms of their suitability for biotechnological applications.

The products of the PAD—phenylacetic acids and derivatives thereof—are industrially very important because they are often employed in different fields. They are applied as flavors and fragrances in honey and wax [17, 18]. Moreover, they are used as precursors of cosmetic [19] and pharmaceutical products. 4-Methyl phenylacetic acids are applied for production of drugs against cancer [20, 21], 4-fluoro phenylacetic acids for medicine used during disorder of gastrointestinal tract, bladder, and nervous system [22]. There are numerous other areas of applications for phenylacetic acids but the probably best known are both analgetics Ibuprofen (4-isobutyl-α-methylphenylacidic acid; CAS 15687-27-1) [23] and Diclofenac (2-(2,6-dichloroanilino)phenylacetic acid; CAS 15307-86-5) [24]. Nowadays, phenylacetic acids are produced by numerous chemical syntheses which are often harmful to the environment [17, 25, 26]. Therefore, the biotechnological production of can be an eco-friendly alternative. The precondition for novel phenylacetic acid synthesis routes are detailed studies about the required enzymes like PADs.

In this work, we especially studied PADs of the styrene-degrading soil bacteria *Rhodococcus opacus* 1CP, *Gordonia rubripertincta* CWB2, and *Sphingopyxis fribergensis* Kp5.2. Moreover, we executed further experiments towards the recently characterized FeaB of *E. coli* K-12 [13]. Understanding the function and applicability of those enzymes will allow to add it towards enzymatic cascades producing valuable phenylacetic acid derivatives. In order to choose a proper candidate PAD, we need to know more about their activity and stability which were investigated herein.

## Materials and Methods

### Chemicals, Kits, and Plasmids

All chemicals used were purchased from AppliChem, Carl Roth, Fluka, Merck, Riedel-de-Haën, Sigma-Aldrich, and VWR International. Both kits, GeneJET Plasmid Miniprep and CloneJET PCR Cloning, were acquired from Thermo Fisher Scientific, whereas the innuPREP Gel Extraction Kit from Analytik Jena was applied. The expression vector pET16bP was handled as described earlier [27].

### Bacteria, Cultivation, Expression Conditions, and Cell Harvesting

The styrene-degrading bacterium *Sphingopyxis fribergensis* Kp5.2 (DSM 28731, accession number SAMN02872841) [28] was cultivated with mineral media (Brunner, DSMZ medium 462) at pH 6.9 with 100 mM glucose at 30°C. The cloning strain *Escherichia coli* NEB5α (New England Biolabs) and the expression strain *Escherichia coli* BL21 (DE3) pLysS were cultivated with LB media at pH 7.1 and with 100 μg mL^-1^ ampicillin (*E. coli* NEB5α) or with 100 μg mL^-1^ ampicillin together with additionally added 35 μg mL^-1^ chloramphenicol (*E. coli* BL21 (DE3) pLysS) for 14-18 h at 37°C. In case of a pre-cultivation on solid medium, 15 g L^-1^ agar were additionally added. Liquid cultures were incubated under constant shaking at 120 rpm in baffled flasks or tubes. The cultivation and cell harvesting of *Escherichia coli* BL21 (DE3) pLysS (pET16bP+*feaB*-K-12) as well as the FeaB-K-12 preparation were performed as described earlier [13].

To obtain a maximum yield of recombinant protein, expression studies with different media (pre-cultures: LB at pH 7.1, inoculation: 1% (v/v) of pre-culture, main cultures containing 10 mL of LB, LBNB, DYT, TB at pH 7.1), expression durations (6 h, 12 h, 18 h, 24 h), and temperatures (22 °C, 30 °C, 37 °C) were performed for FeaB-Kp5.2 and both StyDs.

The subsequent main cultures for the final expressions contained 800 ml DYT medium at pH 7.1 (FeaB-Kp5.2) and 1000 ml TB medium at pH 7.1 (both StyDs) with the abovementioned antibiotics. For FeaB-Kp5.2 expression, 1% (v/v) of the pre-cultures in TB medium at pH 7.1 was directly used for inoculation. For StyD expression, 1% (v/v) of the pre-cultures containing LB medium at pH 7.1 was centrifuged (5000 *x g*, 4 °C, 5 min) and washed once with 25 mM phosphate buffer (pH 7.3) to remove the medium ingredients. The pellets obtained after subsequent centrifugation (5000 *x g*, 4 °C, 5 min) were resuspended in 10 ml fresh TB medium at pH 7.1, and cell suspensions were added to the main cultures. Until an OD_600_ of about 0.4–0.5 was reached, all cultures were incubated at 37 °C. Afterwards, 0.1 mM isopropyl-ß-D-thiogalactopyranoside was added, and the incubation was continued at the optimized conditions for every protein as mentioned in the “Results” section.

The harvesting of the cultures was performed at 5000 x *g* and 4 °C for 20 min. Afterwards, the pellets were washed once in 25 mM phosphate buffer (pH 7.3) and centrifuged (5000 x *g,* 4 °C*,* 30 min) again. The cells were resuspended in the same buffer and stored at −20 °C until cell disruption.

### Cloning of *feaB* and Gene Synthesis of *styD* Genes

The wildtype gene *feaB-*Kp5.2 (AJA07149.1; sequence attached in supplemental material) originating from strain *S. fribergensis* Kp5.2 was amplified by PCR with the specific primers FeaB-Kp52-fw 5’-CATATGGCAACGGCGCAATCCTAC-3’ and FeaB-Kp52-rev 5’-GCGGCCGCTTAGTGCGCGATGCACACG-3’. The reaction mixture with a final volume of 50 μL consists of 5-μL 10x Dream Taq buffer; 0.5 μM of each primer; 0.08 mM of dATP, dTTP, dCTP, dGTP; and 25 ng extracted genomic DNA of *S. fribergensis* Kp5.2 as well as 2.5 U of DreamTaq polymerase. The PCR was performed corresponding to DreamTaq polymerase instruction (Thermo Fisher Scientific), and an annealing temperature of 52.0 °C was adjusted. The subsequent steps of cloning and transforming were carried out as described in an earlier study [13]. For revision of the cloning success, the cloning product was restricted by NotI as well as NdeI, and the restriction product was subjected to a gel electrophoresis.

The wildtype genes *styD*-1CP-W (KT923291) originating from *R. opacus* 1CP (VKM Ac-2638) [29] and *styD*-CWB2-W (KT923294) originating from *G. rubripertincta* CWB2 (DSM 46758) [30] were optimized using the online tool OPTIMIZER [31]. The codon usage of *Acinetobacter* sp. ADP1 was selected. Restriction sites, which were selected for subsequent cloning, were prevented inside the gene sequence and attached on the flanks of the gene. The optimized gene sequences for *styD*-1CP (KT923292, Gene sequence 2 in Supplemental Material section) and *styD*-CWB2 (KT923295, Gene sequence 3 in Supplemental Material section) were synthesized and ligated into the vector pET16bP by MWG Eurofins. The expression system pET16bP provides a 10-Histidine tag at the *N*-terminal site [27].

The vectors containing the genes of interest were transformed into *E. coli* BL21 (DE3) pLysS and cultivated as mentioned above.

### Preparation of PAD

For further studies towards FeaB-K-12, the already existing expression strain *E. coli* BL21 (DE3) pLysS (pET16bP+*feaB*-K-12) was used and cultivated as described earlier [13].

For the preparation of the three recombinantly produced PADs FeaB-Kp5.2, StyD-1CP, and StyD-CWB2, the appropriate *E. coli* BL21 (DE3) pLysS strains were cultivated and harvested as mentioned above. 40 U DNase I were added to the thawed cell suspensions. Then, the disruption was performed by three passages through a cooled French press at 1500 psi. The crude extract was centrifuged at 50,000 x *g* and 4 °C for 1 h to separate the insoluble cell components from the soluble protein fraction. Afterwards, the soluble proteins obtained were subjected to protein purification by means of Ni-affinity chromatography via fast protein liquid chromatography (FPLC) as described elsewhere [27]. First, the supernatant was loaded, and unspecific proteins were removed with washing buffer (300 mM NaCl, 20 mM Tris-HCl, pH 7.5). To elute the proteins bounded, an elution buffer (300 mM NaCl, 20 mM Tris-HCl, pH 7.5, 500-mM imidazole) was used and a linear gradient of imidazole from 25–500 mM was adjusted. The fractions were screened for dehydrogenase activity using the below-mentioned assay, and active fractions were pooled. In case of FeaB-Kp5.2, the protein preparation was subjected to dialysis over night as described earlier [32], and finally, an equal volume of 99% glycerol (v/v) was added for storage at −20 °C. Both StyDs were transferred from elution to storage buffer via ultrafiltration using Vivaspin centrifugal concentrators (Vivaproducts, Inc.). Therefore, the sample was centrifuged at 4 °C and 5000 *x* g until a residual volume of 1 mL. Afterwards, phosphate buffer (pH 7.7) was added and centrifuged again. The protein solution received was diluted with phosphate buffer (pH 7.7) to the initial volume. An equal volume glycerol was added, mixed carefully and stored at −20 °C. The success of all expression studies and purification procedures was monitored via sodium dodecyl sulfate polyacrylamide gel electrophoresis (SDS-PAGE) as mentioned previously [13]. The concentration of enzyme preparation was monitored by Bradford method employing a bovine serum albumin standard as reference [33].

### Biochemical Characterization

The dehydrogenases activity was measured using the two-step enzyme assay at pH 7.7 which was described earlier by Zimmerling and coworkers [13]. The activity measurements were performed spectrometrically (SpectraMax M2e, Molecular Devices) because the formation of phenylacetic acids can be monitored indirectly by NAD(P)H increase at 340 nm. In addition, product formation was verified by RP-HPLC (Dionex Ultimate 3000: pump, autosampler, diode array detector; software: Chromeleon 7; stationary phase: Knauer C18 Eurospher sorbens: pore size 100 Å, particle size 5 μm, column length 125 mm and inner diameter 4 mm; mobile phase: 50% methanol, 50% water with 2 g L^−1^ H_3_PO_4_ , flow rate 0.7 mL min^−1^; injection volume 10 μL; absorbance range 200–300 nm), and on the basis of available standards, the retention volumes and specific spectra were verified. Applying this assay, styrene oxides are completely transformed into corresponding phenylacetaldehydes (PA) during the first step by enriched SOI-1CP [13]. In the second step, the aldehydes obtained can be used as putative substrates for dehydrogenases. Thus for simplification, it is spoken of (substituted) phenylacetaldehyde as substrate in this publication although the corresponding styrene oxides—completely transformed by the SOI-1CP into the PA before the start for the dehydrogenase activity measurements—were used expect α-methyl phenylacetaldehyde. The following enzyme amounts were applied: 2.8–3.4 ng FeaB-K-12, 1.6–29 ng FeaB-Kp5.2, 37–119 ng StyD-1CP, and 9.2–115 ng StyD-CWB2, respectively. First, the Michaelis-Menten kinetics for NAD^+^ (FeaB-Kp5.2: 0- 10 mM; StyDs: 0–8 mM; PA standard concentration: 0.5 mM) were investigated. The consequential suitable NAD^+^ concentrations (Table 1) were applied to study Michaelis-Menten kinetics for PA (FeaB-Kp5.2: 0.1–1.0 mM; StyD-1CP: 0–1.5 mM; StyD-CWB2: 0.005–0.1 mM). Moreover, the identification of the most suitable cofactor was studied applying 0.5 mM phenylacetaldehyde and the following cofactor concentrations for the three enzymes: 2.5 mM NAD^+^ and NADP^+^ as well as 2.5 mM phenazine methosulfate (PMS) (FeaB-Kp5.2), 6.0 (StyD-1CP) and 2.0 mM NAD^+^ (StyD-CWB2), 5.0 mM NADP^+^ (both StyDs). Furthermore, the ability of all four dehydrogenases to convert different substituted phenylacetaldehydes (4-chloro-, 4-fluoro-, α-methyl-) was studied by applying 0.5 mM substrate and the most suitable NAD^+^ concentration for each dehydrogenase (Table 1).

**Table 1:** Values of Michaelis-Menten kinetics and the turnover number k_cat_(app) for FeaBs and StyDs towards NAD^+^ and phenylacetaldehyde

Enzyme	Substrate (varied) [mmol L^-1^]	Substrate (fixed) [mmol L^-1^]	V_max_(app)^a^ [U mg^-1^]	K_M_(app)^a^ [mmol L^-1^]	k_cat_(app) [min^-1^]	Reference
FeaB-Kp5.2	NAD^+^: 0–10.0	PA: 0.5	17.8 ± 2.1	0.5 ± 0.3	3752	This study
	PA: 0–1.0	NAD^+^: 2.5	14.8 ± 0.3	0.022 ± 0.005	3119	
StyD-1CP	NAD^+^: 0–8.0	PA: 0.5	0.039 ± 0.001	0.38 ± 0.06	9	This study
	PA: 0–1.5	NAD^+^: 6.0	0.095 ± 0.002	0.04 ± 0.01	20	
StyD-CWB2	NAD^+^: 0–8.0	PA: 0.5	0.047 ± 0.002	0.08 ± 0.02	10	This study
	PA: 0–0.1	NAD^+^: 2.0	0.12 ± 0.02	0.06 ± 0.02	25	
FeaB-K-12	NAD^+^: 0-8.0	PA: 0.5	6.5 ± 0.2	0.50 ± 0.05	1396	[13]
	PA: 0–1.25	NAD^+^: 5.0	6.7 ± 0.1	0.018 ± 0.004	1439	

^a^Data shown are averages of independently measured triplicates

Other important characteristics, especially with regard to biotechnological applications, are the behavior of the enzyme after storage at −20 °C for about 3 months as well as the enzyme’s temperature stability over a range of −20 up to 55 °C. The behavior of FeaB-Kp5.2 towards these special conditions was studied by applying the most applicable NAD^+^ (2.5 mM) and standard PA (0.5 mM) concentration. StyD-CWB2 was also tested for protein stability at −20 °C overtime using 2.0 mM NAD^+^ and 0.5 mM PA.

Besides the activity to oxidize (phenylacet)aldehydes to the corresponding acids, formerly studied dehydrogenases showed an esterase activity, too [10, 32, 34, 35]. This conversion can be screened by a photometric method. Thereby, enzymes catalyze the hydrolysis of *p*-nitrophenyl acetate to acetic acid and *p*-nitrophenol. The increase of the last-mentioned product can be easily measured at 400 nm. The esterase activity of the four dehydrogenases was tested by using the assay described previously [10] under application of the optimal NAD^+^ concentration of each enzyme. We applied 20 μL of 2.5 mM *p*-nitrophenyl acetate in 1000-μL assay at pH 7.7. Furthermore, we determined the molar extinction coefficient under these conditions.

## Results

### Identification of Phenylacetaldehyde Dehydrogenases among Soil Bacteria

**Fig. 1:**
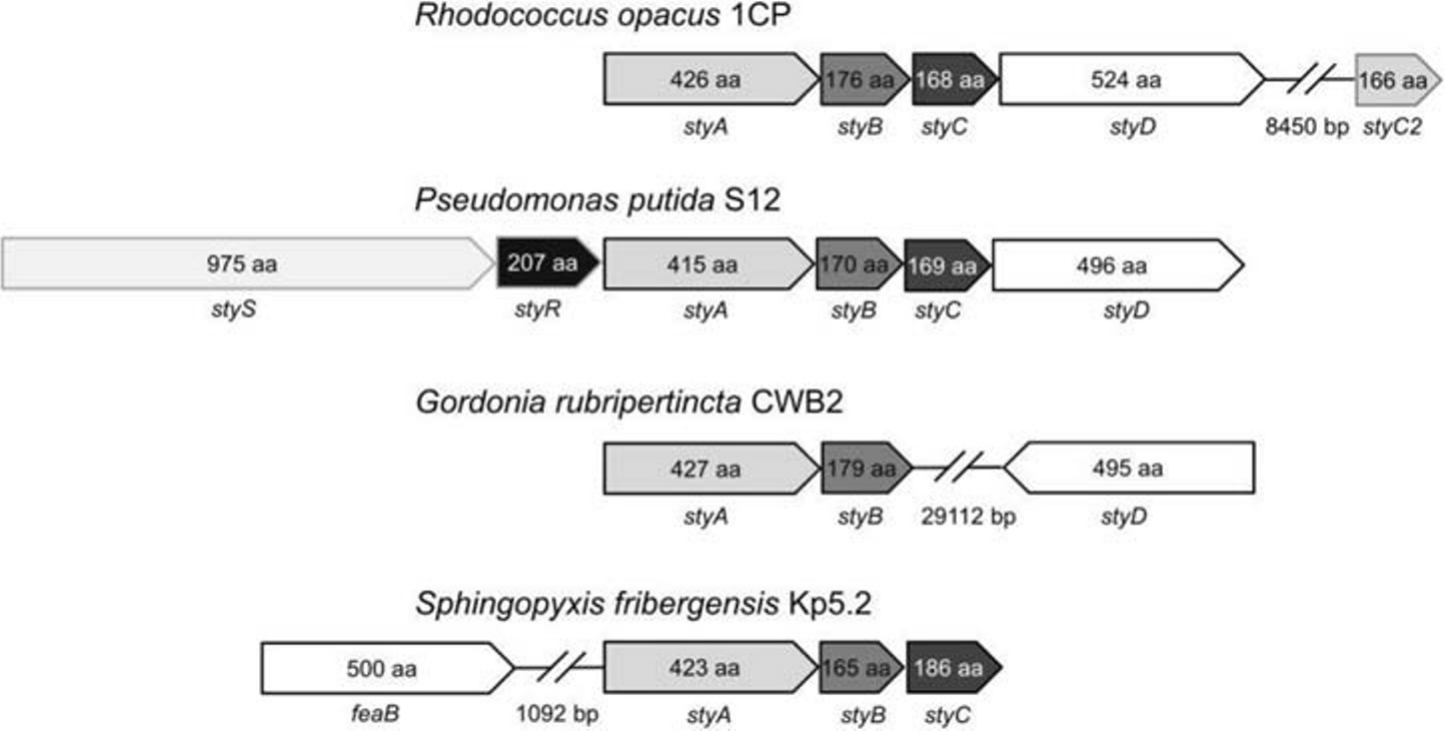
Comparison of the *sty* genes organization of *Rhodococcus opacus* 1CP (CP009112) [39], *Pseudomonas putida* S12 (CP009975) [2], *Gordonia rubripertincta* CWB2 (CP022580) [36], and *Sphingopyxis fribergensis* Kp5.2 (CP009122) [38]. The genes involved in the upper styrene metabolism are *styA* and *styB* (encode the SMO), *styC* (encodes the SOI), *styD*, and *feaB* (encode the PAD), respectively. The gene *styC2* encodes a protein similar to StyC, and the protein products of *styS* and *styR* are a sensor and a regulator proteins [38]

A phenylacetaldehyde dehydrogenase from *Pseudomonas putida* S12 [2] was used as start to genome mining approach for *styD* or *feaB* genes in well-known styrene degrading bacteria [8, 11, 28, 36–38]. As candidate strains, we had chosen *R. opacus* 1CP, *S. fribergensis* Kp5.2, and *G. rubripertincta* CWB2 as those have been determined to provide different organizations in their styrene-degrading properties. This is reflected by different organizations of genes relevant for the upper styrene degradation pathways in respective soil bacteria (Fig. 1). The *sty* gene organization partially differs between the studied soil microorganisms. *R. opacus* 1CP [39] and *P. putida* S12 [2] harbor the “classic” *sty* operon with the order of the *sty* genes identically to the enzymatic cascade—the SMO is encoded by *styA* and *styB*, the SOI by *styC*, and the PAD by *styD*. However, in the genome of *G. rubripertincta* CWB2 *styD* is located far away and upstream from the *styA* and *styB*, and no *styC* could be found [36]. *S. fribergensis* Kp5.2 owns *styA*, *styB*, and *styC* but not *styD*. Indeed, a gene belonging originally to the phenylethylamine degradation called *feaB* is located upstream the *styABC* operon [11, 38]. This gene product FeaB-Kp5.2 acts like the StyD proteins of the other bacteria.

In contrast to the other bacteria, *R. opacus* 1CP has additionally a SOI-like protein StyC2 [38], and *P. putida S12* harbors two regulatory proteins StyS (AJA17111) and StyR styR (AJA17112) directly in front of *styABCD*.

The comparison of the PAD nucleotide sequences shown in Fig. 1 revealed the maximum identity of 77% between StyD-CWB2 and StyD-1CP, by far. All other protein sequences have identities in the range of 44–47% to each other.

### Cloning, Gene Synthesis, and Expression Studies

For revision of the cloning success of *feaB*-Kp5.2, the cloning product was restricted by specific restriction enzymes (NotI, NdeI), and a gel electrophoresis was performed. We gained fragment bands in the expected size of 1500 bp (not shown). Therefore, the successful cloning of *feaB*-Kp5.2 (Gene sequence 1, Supplemental Material) by PCR using specific primers could be verified. The genes *styD*-1CP (Gene sequence 2, Supplemental Material) and *styD*-CWB2 (Gene sequence 3, Supplemental Material) were optimized with respect to the codon usage, synthesized, and verified via standard single strand sequencing by MWG Eurofins (not shown). Finally, the genes of interest were ligated into the expression vector pET16bP and cloned into the expression strain *E. coli* BL21 (DE3) pLysS as described previously for other genes [13, 27]. The StyD and FeaB expression strains obtained were subjected to gene expression studies to gain maximum amounts of active proteins. The highest amount of enzyme was formed at 22 °C and in TB medium at pH 7.1 after 24 h (StyD-1CP), 6 h (StyD-CWB2), or in DYT medium at pH 7.1 after 20 h (FeaB-Kp5.2), respectively. Therefore, these optimized conditions were used for all following gene expression experiments in order to produce proteins for subsequent studies.

### Protein Enrichment by FPLC and Verification by SDS-PAGE

The recombinantly produced His_10_-tagged proteins FeaB-Kp5.2 and FeaB-K-12 as well as StyD-1CP and StyD-CWB2 were enriched by FPLC as mentioned above. The proteins eluted at the following imidazole concentration in the below-mentioned volume: FeaB-Kp5.2 in 16 mL at 175–410 mM, StyD-1CP in 8 mL at 260 mM, and StyD-CWB2 in 12–16 mL at 425–500 mM imidazole. The protein fractions obtained were investigated for phenylacetaldehyde dehydrogenase activity applying the two-step enzyme assay [13]. Active protein fractions were diluted with the same volume of glycerin (99%) and stored as described previously [13].

The success of enrichment via FPLC and all expression studies was monitored by SDS-PAGE. The enzyme-containing cell suspension, the supernatant after separation from disrupted cells as well as the FPLC fractions were controlled (Fig. S1). In the case of FeaB-Kp5.2 and StyD-CWB2, a complete purification could be proven while the enzyme from strain 1CP was considerably purified. The protein concentrations of the pooled and pure FPLC fractions were measured using the Bradford method as described in “Materials and Methods” section. The optimized conditions for PAD expression led to a high amount of active enzyme for the following characterization experiments. We obtained up to 4.8 mg StyD-CWB2 in 2.0 L, 2.6 mg StyD-1CP in 1.8 L, and 27.9 mg FeaB-Kp5.2 in 1.6 L culture volume, respectively.

### Biochemical Characterization

**Fig. 2:**
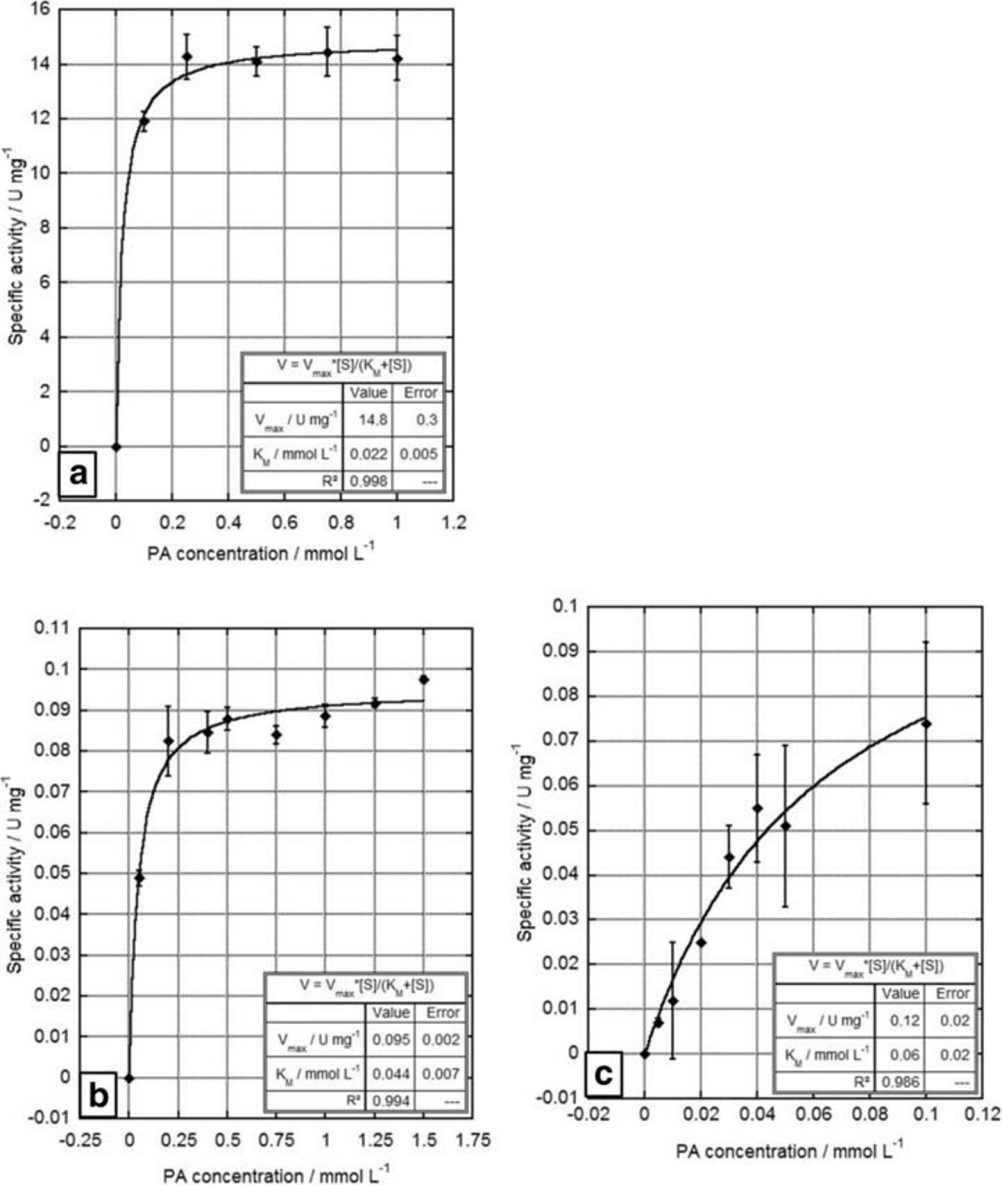
Michaelis-Menten plots of (**a**) FeaB-Kp5.2, (**b**) StyD-1CP, and (**c**) StyD-CWB2 activities using different concentrations of phenylacetaldehyde. The assay was performed as mentioned in the “Materials and Methods” section. Various PA concentrations 0–1.0 (FeaB-Kp5.2), 0–1.5 (StyD-1CP), and 0–0.1 mmol L^-1^ (StyD-CWB2) were applied, while the most suitable NAD^+^ concentration (FeaB-Kp5.2: 2.5; StyD-1CP: 6.0; StyD-CWB2: 2.0 mmol L^-1^) was set corresponding to each enzyme. Data shown are averages of independently measured triplicates

The recombinantly produced and purified dehydrogenases FeaB-Kp5.2, StyD-1CP, and StyD-CWB2 were studied regarding their biochemical behavior applying the two-step enzyme assay [13]. Especially, the Michaelis-Menten kinetics for the co-substrate NAD^+^ and the substrate phenylacetaldehyde were investigated (Fig. 2, Table 1). First, the kinetics of NAD^+^ were studied using a constant phenylacetaldehyde (PA) concentration of 0.5 mmol L^-1^. Afterwards, the PA concentrations were varied, and the most suitable NAD^+^ concentration depending on the enzyme (FeaB-Kp5.2: 2.5; StyD-1CP: 6.0; StyD-CWB2: 2.0 mmol L^-1^) was used (Fig. 2). FeaB-Kp5.2 showed maximum reaction rates (V_max_(app)) of 17.8 ± 2.1 U mg^-1^ for NAD^+^ and 14.8 ± 0.3 U mg^-1^ for PA. For StyD-1CP and StyD-CWB2, the V_max_(app) values of NAD^+^ kinetic were 0.039 ± 0.001 U mg^-1^ and 0.047 ± 0.002 U mg^-1^. Remarkably, the activities for PA kinetic were about 2.5 times higher (0.095 ± 0.002 U mg^-1^ for StyD-1CP and 0.12 ± 0.02 U mg^-1^ for StyD-CWB2) than those for the co-substrate.

Furthermore, the behavior of three PADs was studied comparing the enzyme activities towards NAD^+^ and NADP^+^. In the case of FeaB-Kp5.2, PMS was also investigated as potential co-substrate (Table 2). This study revealed the same tendency for all enzymes. The most suitable cofactor was NAD^+^. The following enzyme activities could be measured for a standard biotransformation using NAD^+^: 13.9 ± 0.7 U mg^-1^ (FeaB-Kp5.2), 0.068 ± 0.001 U mg^-1^ (StyD-1CP), and 0.0356 ± 0.0001 U mg^-1^ (StyD-CWB2). For NADP^+^, the residual activities were 0.6 ± 0.2 U mg^-1^ (FeaB-Kp5.2), 0.020 ± 0.001 U mg^-1^ (StyD-1CP), and 0.011 ± 0.001 U mg^-1^ (NADP^+^) comparing the enzyme activities towards NAD^+^. PMS did not serve as co-substrate for FeaB-Kp5.2.

**Table 2:** Dehydrogenase activities^a^ of FeaBs and StyDs depending on co-substrate

Dehydrogenase	NAD^+^ [U mg^-1^]	NADP^+^ [%]	PMS [%]	Reference
FeaB-Kp5.2	13.9 ± 0.7	4.0 ± 1.2	n.a.	This work
StyD-1CP	0.068 ± 0.001	29.5 ± 1.6	n.m.	This work
StyD-CWB2	0.0356 ± 0.0001	31.7 ± 2.5	n.m.	This work
FeaB-K-12	3.1 ± 0.2	10.6 ± 0.3	n.a.	This work
PadA	2.66	16 fold less	n.m.	[10]

^a^Data shown are averages of independently measured triplicates

n.a. no measurable activity

n.m. not measured

Moreover, the three dehydrogenases were studied towards their capability to convert substituted phenylacetaldehydes in comparison with the non-substituted substrate (Table 3). It is very noticeable that FeaB-Kp5.2 showed activities of 10.7 ± 0.6 U mg^-1^ (phenylacetaldehyde), 10.7 ± 1.2 U mg^-1^ (4-chlorophenylacetaldehyde), 9.6 ± 0.4 U mg^-1^ (4-fluorophenylacetaldehyde), and 1.0 ± 0.1 U mg^-1^ (α-methylphenylacetaldehyde), whereas the enzyme activities of both StyDs are located only in the range of several mU mg^-1^. StyD-1CP converted phenylacetaldehyde with 0.044 ± 0.002 U mg^-1^, and the activity towards 4-fluorophenylacetaldehyde was half as high (0.021 ± 0.001 U mg^-1^). The least suitable substrates were the methylated (0.0155 ± 0.0003 U mg^-1^) and chloro-substituted ones (0.009 ± 0.001 U mg^-1^). In comparison to all other dehydrogenases described here, the most suitable substrate for StyD-CWB2 is 4-fluorophenylacetaldehyde (0.030 ± 0.001 U mg^-1^), followed by the non-substituted aldehyde (0.0256 ± 0.0005 U mg^-1^) and α-methylphenylacetaldehyde (0.016 ± 0.001 U mg^-1^). This enzyme showed the lowest activity of 0.008 ± 0.001 U mg^-1^ towards the chloro-substituted substrate. To complete the results of our recent study [13], the enzyme activity of FeaB-K-12 towards α-methylphenylacetaldehyde with 1.6 ± 0.2 U mg^-1^ was investigated, too.

**Table 3:** Enzyme activities^a^ of FeaBs and StyDs towards non-substituted and three substituted phenylacetaldehydes

Dehydrogenase	Dehydrogenase activities^a^ towards SOI-1CP produced substrates*** [U mg^-1^_protein_]				Reference
	Phenylacetaldehyde	4-Chlorophenylacetaldehyde	4-Fluorophenylacetaldehyde	α-Methylphenylacetaldehyde	
FeaB-Kp5.2	10.7 ± 0.6	10.7 ± 1.2	9.6 ± 0.4	1.0 ± 0.1	This work
StyD-1CP	0.044 ± 0.002	0.009 ± 0.001	0.021 ± 0.001	0.0155 ± 0.0003	This work
StyD-CWB2	0.0256 ± 0.0005	0.008 ± 0.001	0.030 ± 0.001	0.016 ± 0.001	This work
FeaB-K-12	6.1 ± 0.7*	3.2 ± 0.6**	3.1 ± 0.5**	1.6 ± 0.2**	*This work/ **[13]
FeaB-K-12	< 0.001	n.m.	n.m.	n.m.	[12]

^a^Data shown are averages of independently measured triplicates

***expecting α-methyl-phenyl-acetaldehyde

n.m. not measured

**Fig. 3:**
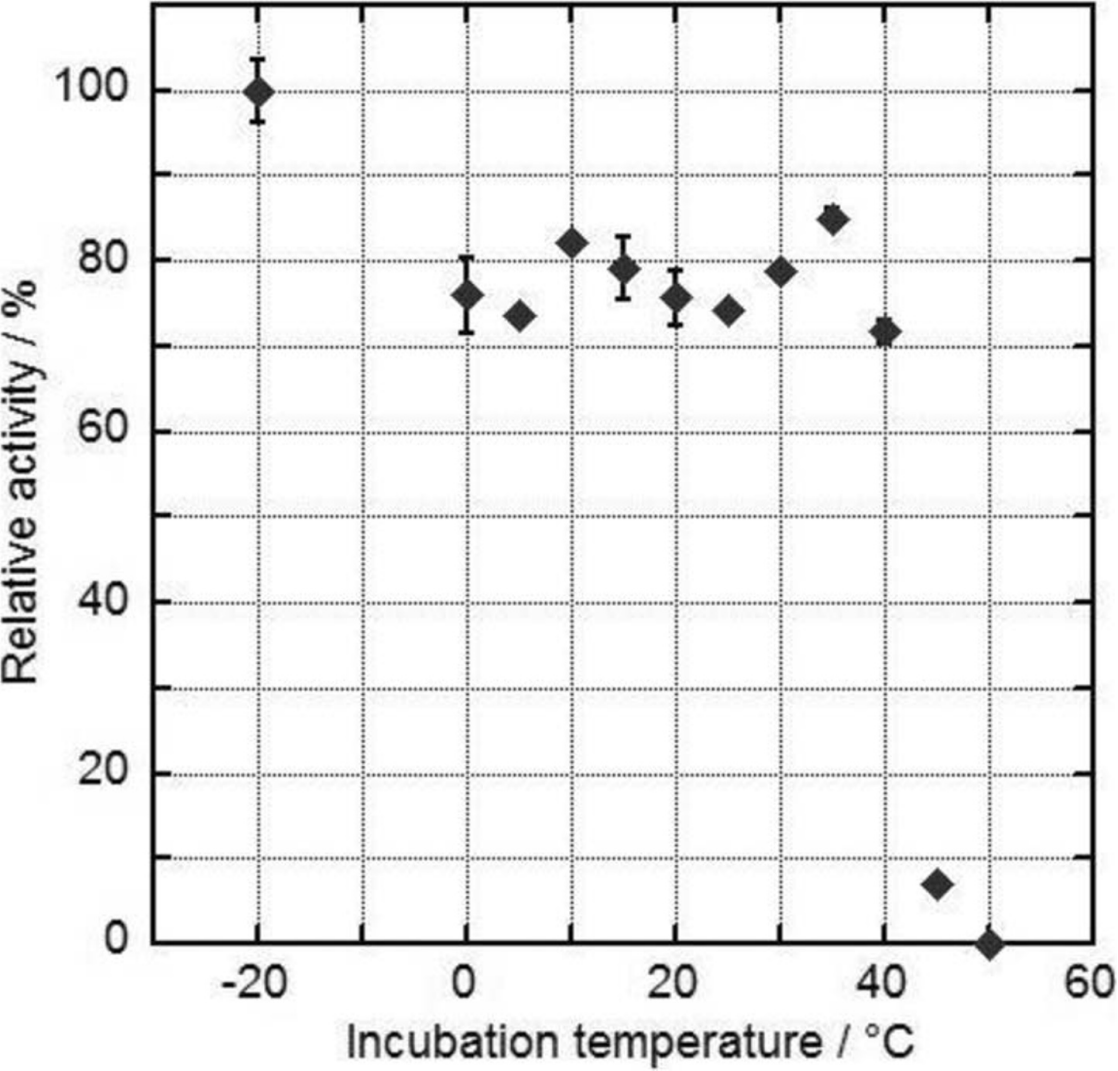
Relative residual activities of FeaB-Kp5.2 after incubation at temperatures between 0 and 50 °C in steps of 5 °C. The assay was performed as mentioned in the “Materials and Methods” section. The enzyme was heated up at the desired temperature and incubated for 30 min before it was investigated by the assay. Relative activities calculated by comparing of the residual activities to the initial activity (value at −20 °C; 8.4 0.3 U mg^-1^) of the enzyme preparation are given. Data shown are averages of independently measured triplicates

For FeaB-Kp5.2 and StyD-CWB2, the activities after 3 months of storage at conditions as mentioned in the “Materials and Methods” section were studied, and a huge difference could be observed. While StyD-CWB2 showed 92.3 ± 3.9% of the initial activity after 93 days, the remaining activity in the case of FeaB-Kp.5.2 was only 44.3 ± 1.6% after 76 days. For the considerably higher active enzyme FeaB-Kp5.2, a study investigating the temperature stability was additionally performed. Therefore, the enzyme was incubated for 30 min at 0–50 °C in steps of 5 °C, and the residual activity after this treatment was compared with the initial activity of the enzyme preparation (Fig. 3).

Besides the activity to oxidize (phenylacet)aldehydes to the corresponding acids, formerly studied dehydrogenases showed an esterase activity, too [10, 32, 34, 40]. This ability can be monitored applying an earlier described photometric assay [10]. The measured molar extinction coefficient for our experimental setting was 3.248 mM^-1^ cm^-1^. Regarding the esterase activity, the behavior of the four dehydrogenases studied is very diverse (Table 4). The highest specific activity during the hydrolysis of *p*-nitrophenyl acetate to acetic acid and *p*-nitrophenol could be measured for FeaB-Kp5.2 with 4.1 ± 0.2 U mg^-1^, followed by 2.6 ± 0.5 U mg^-1^ (FeaB-K-12) and 1.4 ± 0.1 U mg^-1^ (StyD-CWB2). By far, the lowest esterase activity of 0.0264 ± 0.0001 U mg^-1^ was detected for StyD-1CP.

**Table 4:** Esterase activities^a^ of FeaBs and StyDs

Dehydrogenase	Specific esterase activity [U mg^-1^]	Relative activity* [%]	Reference
FeaB-Kp5.2	4.1 ± 0.2	86 ± 5.0	This work
StyD-1CP	0.0264 ± 0.0001	60 ± 0.2	This work
StyD-CWB2	1.38 ± 0.09	5391 ± 352	This work
FeaB-K-12	2.6 ± 0.5	144 ± 27	This work
PadA	0.0056	0.3	[10]
BADH-Pp	1.94	13	[35]

^a^Data shown are averages of independently measured triplicates

*In comparison with dehydrogenase activity towards the standard substrate phenylacetaldehyde

## Discussion

### Phylogenetic Classification of These PADs

**Fig. 4:**
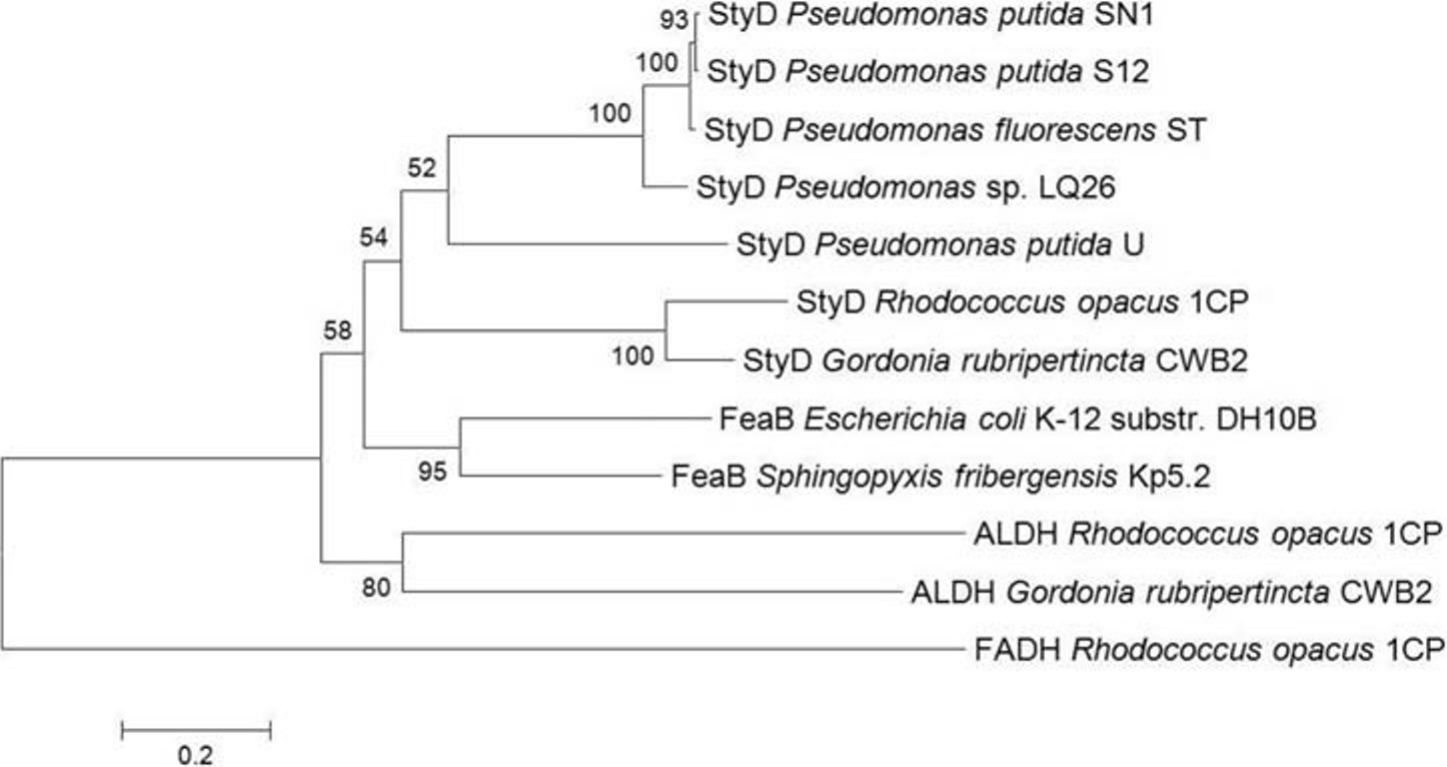
Phylogenetic analysis of various (phenylacetaldehyde) aldehyde dehydrogenases. The phylogenetic tree was calculated with MEGA6 using the minimum evolution method with 1000 bootstrap replications. Different StyDs originating from various pseudomonads as well as StyDs, FeaBs, and ALDHs from *Rhodococcus opacus* 1CP, *Gordonia rubripertincta* CWB2, *Escherichia coli* K-12 substr. DH10B, and *Sphingopyxis fribergensis* Kp5.2 are considered

For visualization of the evolutionary relationship of some already published dehydrogenases and those studied in our former and current research work, a phylogenetic tree (Fig. 4) was calculated with the software MEGA6 using the minimum evolution method and a bootstrap value of 1000. The phylogenetic tree was constructed by using twelve ALDH, one of the family “Class 3 ALDH” and eleven aromatic ALDHs. The fatty aldehyde dehydrogenase (FADH) of *R. opacus* 1CP was used as outgroup based on the “summary tree of ALDH families” shown previously [1]. This enzyme is a representative of family “Class 3 ALDHs” which have no strict substrate specificity and used both cofactors, NAD^+^ and NADP^+^. They are involved in different pathways like the metabolism of lipid peroxidation products and long-chain fatty aldehydes [1]. In our tree, two main branches are visible. The lower branch is formed by both unspecified ALDHs of *R. opacus* 1CP and *G. rubripertincta* CWB2 which had been studied marginal in our former research work [13]. The upper branch is exclusively formed by enzymes belonging to the aromatic ALDHs forming a separate family. Here, they are called FeaB and StyD, respectively. Based on the former study of 145 full-length sequences of aldehyde dehydrogenases, the aromatic aldehyde dehydrogenases have presumable a recent common ancestor because their sequences are closely related to each other [4]. Furthermore, representatives of this family were only found in bacteria and have clear cofactor preferences to NAD^+^, and they oxidize specific aromatic aldehydes into the corresponding acids [1, 41]. This branch is subdivided into two twigs, one contains both FeaB enzymes, and the other is built by seven StyD proteins. Thereby, both StyDs originating from *R. opacus* 1CP and *G. rubripertincta* CWB2 seem to be more related to each other in comparison with all other StyDs from the pseudomonads. This assumption is also confirmed by the sequence identities presented in the “Results” section, chapter “Identification of phenylacetaldehyde dehydrogenases among soil bacteria”.

This evolutionary pattern is confirmed by the results of enzyme characterization studies. The enzyme activities of both FeaBs were almost in the same range and obviously distinguished from those of the two StyDs (Table 1, Table 2, Table 3). Both previously characterized ALDHs originating from *R. opacus* 1CP and *G. rubripertincta* CWB2 [13] behaved similar to each other and, however, pretty different compared with the FeaBs and StyDs described in the present study. The clustering of the various pseudomonas’ StyDs was created by the alignment of the protein sequences. They seem to be quite related to each other. However, statements about the biochemical properties and presumptive similarities cannot be done because of a lack of sufficient data for these enzymes.

### Biochemical Characterization

The three dehydrogenases FeaB-Kp5.2, StyD-1CP, and StyD-CWB2 were recombinantly produced and enriched by Ni-affinity chromatography for detailed biochemical studies. First of all, it must be said that all showed results are activities not of the native but the His_10_-tagged proteins. In former studies, the influences of various His-tags on the N- and C-termini were investigated [42–49]. On basis of those research works, no uniform statement can be done about the influence of His-tags. Presumably, it depends on the nature of the enzyme, the His-tag variant, and location. These investigations tend to result that the His-tags have no or a positive effect on the enzymes’ activity [42, 44, 45]. Hence, our findings cannot be deputed on the native dehydrogenases because we studied exclusively the recombinant proteins. Nevertheless, our results about the characterization of the investigated dehydrogenases add valuable knowledge to the field as discussed below.

At the beginning, the Michaelis-Menten kinetic for the co-substrate NAD^+^ was investigated. The results for this study were very different between the FeaBs and StyDs (Table 1). FeaB-Kp5.2 showed a significantly higher enzyme activity. The V_max_(app) value was 379 and 456 -times higher than those of the StyDs. Compared with a recent study, V_max_(app) of FeaB-Kp5.2 is in the same range than this of FeaB-K-12 [13]. Considering the K_M_(app) value, FeaB-Kp5.2 and both StyDs behave quite different in comparison to FeaB-K-12 [13]. This study revealed the suitable NAD^+^ concentration for these enzymes which were applied for all following investigations.

Following this, the Michaelis-Menten-kinetic for the substrate phenylacetaldehyde (PA) was tested (Fig. 2). For both StyDs, V_max_(app) values of PA kinetics in combination with the suitable NAD^+^ concentration were about 2.5-times higher than those of the co-substrate, but also considerably lower compared with the values measured for the FeaB enzymes. Considering all V_max_(app) values, both FeaBs are the much more active compared with the investigated StyDs. As expected, this is supported by the corresponding k_cat_(app) values (Table 1). Under these experimental conditions, there are tremendous differences between the FeaBs and StyDs. The lowest turnover numbers of about 7 and 9 were reached for StyD-1CP and StyD-CWB2, respectively. In contrast, both FeaBs reached significantly higher k_cat_(app) values of 709 (FeaB-K-12) and 1718 (FeaB-Kp5.2). In summary, the FeaB-related enzymes exceed the turnover number of the StyDs by roughly 100–240 times.

Aldehyde dehydrogenases are necessarily addicted to co-substrate due to their mechanism [2]. Here, we tested NAD^+^, NADP^+^, and PMS as electron acceptor (Table 2). Interestingly in this characteristic, all four investigated enzymes showed the same preference. The enzyme activity by applying NAD^+^ was the highest, by far. Using these activity values as reference, the residual activities during phenylacetaldehyde transformations with the co-substrate NADP^+^ under standard conditions were only 31.7 ± 2.5 (StyD-CWB2), 29.5 ± 1.6 (StyD-1CP), 10.6 ± 0.3 (FeaB-K-12), [13] and 4.0 ± 1.2 % (FeaB-Kp5.2). In analogy to the previously characterized FeaB-K-12 [13], we tested the capability of PMS as co-substrate for FeaB-Kp5.2. Indeed, there was no measureable enzyme activity by applying PMS, too. Respectively, it can be concluded that phenylacetaldehyde dehydrogenases even if they originate from different pathways are NAD^+^-dependent and rather specific for this co-substrate. In case of the degradation of styrene, this makes sense as here the first enzyme of this pathway, the styrene monooxygenase (StyAB), utilizes NADH and thus provides NAD^+^ which could be used by StyDs or FeaBs. This is reported as a natural intrinsic enzyme cascade [8].

Moreover, all three dehydrogenases were studied towards their capability to convert substituted phenylacetaldehydes in comparison with the non-substituted one (Table 3). FeaB-Kp5.2 was able to metabolized 4-chlorophenylacetaldehyde as good as phenylacetaldehyde. Residual activities of 90 ± 4 and 9 ± 1% were detected towards 4-fluoro- and α-methylphenylacetaldehyde. As expected, both FeaBs behave similar in this context. They showed the highest relative enzyme activities in the presence of the non-substituted phenylacetaldehyde and the lowest during α-methylphenylacetaldehyde conversion. Regarding to the halogenated substrates, the enzyme activities of FeaB-K-12 were obviously lower [13]. During the α-methylphenylacetaldehyde oxidation, both FeaBs showed nearly the same absolute enzyme activity. So, this is the only substrate which is metabolized slightly faster by FeaB-K-12 instead of FeaB-Kp5.2. For the other three substrates, FeaB-Kp5.2 showed about two times higher transformation rates in comparison with FeaB-K-12. However, both StyD proteins showed completely different characteristics. In the case of StyD-1CP, the non-substituted phenylacetaldehyde was the best substrate, whereas this enzyme converted 4-fluoro- and 4-chlorophenylacetaldehyde with relative activities about half and one-fifth. StyD-CWB2 acted as an exception because the highest activity of 116 ± 5% in comparison with phenylacetaldehyde was measured in the presence of 4-fluorophenylacetaldehyde. Towards α-methylphenylacetaldehyde, StyD-CWB2 showed the highest relative enzyme activity measured for this substrate, so far. The enzyme from CWB2 showed its lowest activity for 4-chlorophenylacetaldehyde. The reduced capability to transform the chloro-substituted substrate was also determined for StyD-1CP. This aspect indicates a remarkable difference of both StyDs towards the FeaBs. In summary and based on the total activities determined in our study, FeaB-Kp5.2 is the most active enzyme towards phenylacetaldehyde and the halogenated substrates with very high activities of 90–100%, while FeaB-K-12 is the most active enzyme towards α-methylphenylacetaldehyde.

Besides the substrate spectra of these enzymes, the stability regarding to two different aspects was additionally studied: the long-term storage over about 3 months at −20°C and the stability towards temperatures in the range of 0–55 °C (Fig. 3). FeaB-Kp5.2 showed a high stability with a residual activity of 44.3 ± 1.6% compared with the initial activity after 76 days at −20 °C. Surprisingly, the long-term stability of StyD-CWB2 was even higher. That enzyme lost only about 8% of its initial activity after 93 days. Furthermore, the stability of FeaB-Kp5.2 towards temperatures up to 40 °C (Fig. 3) was notable because residual activities of 85–72% were measured after 30-min incubation at 0–40 °C compared with the initial activity at −20 °C. For temperatures between 40 °C (71.7 ± 1.4%) and 45°C, the residual enzyme activity decreased tremendously to 6.9 ± 0.1%. No residual activity could be determined after incubating the enzyme at a temperature of 50 °C indicating a total inactivation of the dehydrogenase. These results for FeaB-Kp5.2 were again similar to those of FeaB-K-12 [13]. Due to the already low enzyme activities for both StyDs, no temperature stability experiments were performed.

ALDHs are known to show dehydrogenase as well as esterase activity. Against former assumptions [50], numerous evidences were found that these enzymes catalyze both distinct reactions in one single active site [51]. These both catalytic functions were first revealed for horse and human liver aldehyde dehydrogenases [40, 52]. Also the bacterial ALDH, PadA from *Escherichia coli* W, was found to have esterase activity [10, 32]. The comparison of the *E. coli* dehydrogenase and esterase activities is hardly possible because the experimental conditions of our and the former study of Ferrández and colleagues (1997) were not similar. However, PadA of *E. coli* W had shown an extremely slight esterase activity of less than 0.3% (5.6 mU mg^-1^) compared with its dehydrogenase activity. In our study, all four tested enzymes showed an esterase activity, too (Table 4). In comparison with the PAD dehydrogenase activity, huge distinctions were determined. Whereas FeaB-K-12 showed a relative esterase activity of 144 ± 27% compared with its dehydrogenase activity, the esterase activities of FeaB-Kp5.2 (86 ± 5%) and StyD-1CP (60.0 ± 0.2%) were lower compared with their dehydrogenase activity (Table 3). Nevertheless, StyD-CWB2 showed a tremendous higher esterase than dehydrogenase activity of 5391 ± 352%. This result could give an assumption of the origin of the StyD originating from *G. rubripertincta* CWB2. Furthermore, this strain has a totally different pathway for styrene [36]. Both aspects strongly indicate that the pathway and the enzymes included were assembled from various other metabolic ways and sources.

### Future Prospectives of These Dehydrogenases

In our study, we have presented entirely novel data about PADs of three styrene-degrading soil bacteria. The results shown differences between these enzymes especially regarding their activities and substrate specificities. So far, FeaB-Kp5.2 could be highlighted as the most active PAD. Hence, we have created a basis of further studies with respect to biotechnological phenylacetic acid syntheses. Therefore, a combination of PAD with SOI and SMO could be practicable for the eco-friendly product of the high-grade phenylacetic acids from simple available styrenes. In contrast to the chemical syntheses, the biotechnological production could be performed under physiological conditions, without plenty of acids and bases and without toxic by-products, too. Hence from an ecological point of view, the development of biotechnological phenylacetic acid synthesis routes is a serious alternative to the current processes.

## Electronic supplementary material

ESM 1 (DOCX 126 kb)
