# Supplementary material for: Biochemical Characterization of Phenylacetaldehyde Dehydrogenases from Styrene-degrading Soil Bacteria
Source: Appl Biochem Biotechnol. 2020 Oct 27;193(3):650–67. doi: 10.1007/s12010-020-03421-8 (PMC7910268; doi:10.1007/s12010-020-03421-8)

# Supplemental Material

Gene sequence 1: *feaB*-Kp5.2 (AJA07149.1; original)

atggcaacggcgcaatcctacgtaaccccgctcagcgcggtctccactttcatcgggcatgatcacgggctcttcatcgatggcgactggcgcgctgcgaagggtaccgaacgacttgacgtcttcgatccctcgaccggagaacccatcgcgagcgtcgccaatgcgtcgcccgacgatgtcgatcatgccgtgcgcgctgcccacaaggcgttcgagagcggcgtctggagcggcattgcccccgccgagagggaacgcctgctcctcaagtttgccgatctggtcgaagccaatgcggaggaactggcgcagatcgaaacgctcaaccagggcaagtcgatcaacatctcgcgtttcgtcgatgtcggtggcgcaccggcttacatgcgttacgtcgctggcctcacgacgaagatcaccggggaaacgttcgacgtctcgatcggcgcgattccaggtgcacgtttcacggcctcgacgcgccgcgaacctatcggcgtcgtgggcgcgatcgcaccgtggaacttcccgatgatgatcgggctgtggaaaatcatgccggcgcttgcagctggctgcaccgtcgtccttaagccatcggaagtcacgccgcttacgagcttgcggcttgcgagtctggcgaaggaggcggggcttcccgcgggtgttctcaacgtgatcaccggtgacggcaaggcaggcaaagcgctggtcgatcatccgctcgtggcgaagatcacgttcaccggttcgaccgcaaccggcaaggcgatcgcgcgatctgcatcggaccggcttttgcgcacctcgctcgaacttggcggcaagaaccccgccatcttccttgccgacgcgcccatcgaacaggcggtccagggtgcaatcctcggcggctttttcaacaacggtcaggtctgcgcggcctcctcgcggctctatatcgcgcgcgccatctatgatcccttcatcaaggctcttgcccacgcggtcgatggtatgaccatgggcccagggctggacacggccgcgcagatcaatcccgtggtttcattacagcaccgcgcgaagattctggaccatatagcgcgcgcgcgtcaggatggcgcccagattgtcgcgggaggtgaaagtcccgaccgcgctggctactatgtccggccgaccgttgtagtcggcgcgggagcgcagcttgccatatcccggcaggaggtcttcgggccgctggtcaccgtcacccccttcgacgatgaggacgaagcgatcaaccttgccaatgataccgaaatgggcctgacggcctcgttgtggactaacgatctcaagaaaacgatggacctcgtaccccggatcaaggccggcacggtctgggtcaattgccacaacttgatcgatcccaatatgccgtttggaggctacaaggaatccgggatcggccgcgacttcggcatccattcgctggacggctatagcgaggtaaagtccgtgtgcatcgcgcactaA

Gene sequence 2: *styD*-1CP (KT923292; codon optimized)

CATATGCCTGAAATTACATTTACGGAACGTATTCGTCGCTTAGGTAGTCGTTTTACAGCGCCACGCCCAACACAGAAGGAATTAGCTATGACTACAGCAGATACATTACCTGGTGTGGGTACACCTCTGCCAGCAGGTACCCCAACCCAACTTTTTTTAAATGGTAACTGGCAACCGGCATCAGATGGTGGTGTATTTACTAACATTAATCCAGCTACCGAACAGGTTCTTGCTGAGGTTTCAGCAGGTACCCCTGATGATATTGACGTTGCGGTCCGTGCCGCGCGTGCACAATTAGGTGGTCAGTGGGGTGCCACTCCTGGTGTAGTTCGTGGTCAGATTCTTAACAAGATCGCTGACCTGATTGAACGTGATATTGATATTCTTGCAACTTTAGAAGCATTGGATGTTGGTAAACCAGTGGGTCAGCCTAAGCTTGACGTTCCAAACGCTGCGGCTACCTTTCGTCATTTCGCTGGCTGGGCAGATAAAATCACCGGCTTTTCGATTCCAACTGCTGGTTATTTCGGTAAACCAACCCATAGTTTTACTGTTCGCGAGCCTGTCGGTGTTATTGGTGCAATTGTTCCTTGGAACACGCCATTGATGATCGCAGCCTGGAAACTTGCTCCAGCATTAGCGTGCGGTAACACAGTAGTTGTAAAACCACCTGAAGAAGCACCATTATCAATCCTACACCTTGGCAAAATTTTGGAAGAAGCGGGTTTACCGGCTGGTGTTGTGAGCATTGTTCCAGGCAACGGTGCTGTTACAGGAAATGCTCTTGTTGAACATCCTGATGTCGATAAAATTTCATTTACCGGTTCTCCAGCAGTTGGTCGTTTAATTCAACAACGTGCGGCTGCGACATTTAAACGTGTAACTCTTGAGCTGGGTGGTAAATCACCGCAAATCATTCTTGATGATGCAGATGTGGAGGCTGCAGTTCAAGGTACTGCAATGGGCCTTTTCTTCAATCAAGGTGAGGTGTGCGCTGCTGGCACTCGTGTTTTCGTACATCGTAGTCGTTACAACGACGTTGTAGATGCATTATCAGGTGCAGCCGATGCACAGGTACTTGGCGATCCATTTGATCCTTCTACAACATTAGGAGCTCTGGTTTCAGCTAAACAGCGTGATACCGTACTATCTTACATCGAACAAGGTAAGAAAGAAGGTGCACGTGTAGCAGCAGGTGGTACACGTCCAGATCATTCTGGTTATTTCGTTCGCCCAACGATCTTTGCTGACGCAAATAATGATATGACTATTGCGCGTGAAGAAATCTTCGGCCCAGTGGGTACGGTTATTGCATTCGATGACCCAGAAGAAGCAATTCGTCTTGCTAATGACACTCAGTACGGTCTTGCTGCTTCTATTTGGACTCGTGACGTATCTCGTGCCCACACTCTAGCACGTCAGGTTCGTGCCGGTGCTGTCTGGGTAAACGGCTGGGCGGCAATTGATCCAGCTTTACCTTGGGGCGGCATGAAAACTAGTGGTATTGGTCGTGAACTGGGCTATGCCGGAATCTTGGCAAACACTGAAGAGAAGGTCGTGACCATTGTGCTTTAGGCGGCCGC

Gene sequence 3: *styD*-CWB2 (KT923295; codon optimized)

CATATGACATCAGTACAAGCGGGCTCTGTTAGCGATTTGTTACCACCAGATACTGCATTGGATCTATTCGTAAACGGTCGTTGGCGCCCAGCTGCTTCTGGCGCAACGTTCGAAGACTTACACCCAGCAACTGGCCAACTGCTAGCACACGTTGCTGCAGGTTCGCCAGAAGACGTAGATGATGCGGTACGCGCAGCTCGCGCACAGCTTGACGGCGAATGGGGTTCAACGTCTGGTGTCACGCGTGGTCAAATTTTACACCGTGTTGCAGACCTTATTGAACGTGATGGTGAAGTTTTGGCACGTTTGGAAGCATTGGATATTGGTAAACCTGTAGGCCAACCTGGCATGTTAGATGTGCCAAATGCAGCCGCAACTTTCCGTCACTTCGCAGGTTGGGCAGATAAAATTACTGGTCAGACTATTCCAACGGCAGGTTACTTTGGTAAACCTACCTTAAGTTACACAGTTCGTGAACCTATTGGTGTAATTGGAGCTATCGTTCCTTGGAATACTCCGTTGATGATTAGCGCTTGGAAATTAGCTCCTGCATTAGCGGCTGGTAATACGGTTGTGGTTAAGCCTCCTGAAGACGCTCCTCTTTCTATTCTTCACCTAGCTCGTTTATTATCTGAAGCTGGTCTTCCTGGTGGCGTTGTGAACGTAGTACCGGGCTTCGGGCACATCGCTGGGGATGCACTTACACGTCATCCGGGAGTCGATAAAATCTCTTTCACCGGTTCTCCACGCGTGGGTAAAATCATTGGTCAAGTTGCTGCTGAAACTTTTAAGCGTGTTACTTTAGAATTAGGTGGTAAATCTCCACAAATCATCTTAGCTGACGCTGATGTTGAGGCAGCAATTAACGGCACAGCGATGGGTCTCTTCTTTAATCAGGGCGAAGTCTGTGCAGCAGGTACACGTGTATTAGTGCATCGTTCTCTTTATGACCAAGTAGTTGATGGTTTGGGTGCAGCTGCAGCTGCTCAAGTGTTGGGTAACCCTCTAGAAGCATCAACTACAATGGGTGCTCTTGTTAATGCAAAACAGCGTGACACTGTTCTGGGCTACATTGAAGCAGGTAAACGAGAAGGTGCGCGTGTTGTAGCAGGTGGTCAAGCAATTGACGGCGACGGTTTTTTCGTTCAACCAACTATTTTTGCAGACGCTAATAATGACATGACAATTGCTCGCGAAGAGATCTTCGGTCCGGTAGGTACAGTAATCCCTTTCGATGATGTTGATGAAGCTATCACTATTGCTAATGATACGACTTACGGCCTTGCTGCTTCGATCTGGACTCGTGACGTTTCTTACGCCCACTCTCTTGCTAAACGTGTACGTGCAGGCGCGGTATGGGTTAACGGGTGGGCTGCAATTGATCCAGCTTTGCCATGGGGTGGGATGAAAACTTCTGGGGTAGGTCGTGAACTGGGTTGGGCGGGTATTGAAGCCAATACTGAGGAGAAGGTTGTTACCGTAGTATTATGAGCGGCCGC

Fig. S 1: SDS-PAGE showing different enrichment and purification steps for StyD-1CP, StyD-CWB2 and FeaB-Kp5.2

The success of the purification of StyD-1CP, -CWB2 and FeaB-Kp5.2 using Ni-affinity chromatography via FPLC was verified by SDS-PAGE. The following samples were electrophoretically laddered: crude extract (CE), insoluble fraction after centrifugation (IF), soluble fraction (SF), discard (D), peak 1 (P1), peak 2 (P2), and peak 3 (P3) of FPLC. The SDS-PAGE showed for all three proteins the correct band at the expected size (StyD-1CP: 57.3, FeaB-Kp5.2: 54.9, and StyD-CWB2: 53.7 kDa). All proteins could be enriched (StyD-1CP) or completely purified (FeaB-Kp5.2, StyD-CWB2) by Ni-affinity chromatography.

Fig. S 1


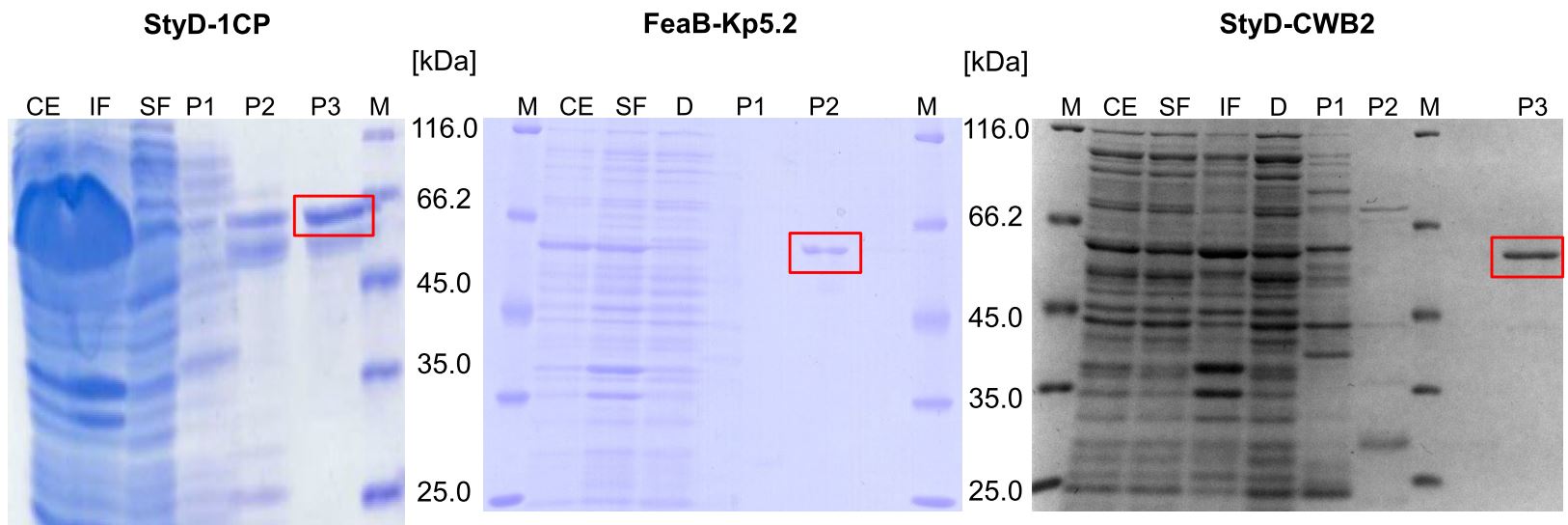

Supplement: Supplementary file 1 — (DOCX 126 kb) [file 12010_2020_3421_MOESM1_ESM.docx]
